# EMD-Based Methodology for the Identification of a High-Speed Train Running in a Gear Operating State

**DOI:** 10.3390/s18030793

**Published:** 2018-03-06

**Authors:** Alejandro Bustos, Higinio Rubio, Cristina Castejón, Juan Carlos García-Prada

**Affiliations:** MAQLAB Research Group, Department of Mechanical Engineering, Universidad Carlos III de Madrid, Av. de la Universidad, 30, 28911 Leganes (Madrid), Spain; hrubio@ing.uc3m.es (H.R.); castejon@ing.uc3m.es (C.C.); jcgprada@ing.uc3m.es (J.C.G.-P.)

**Keywords:** maintenance, high-speed train, vibratory analysis, empirical mode decomposition, EMD, time evolution of spectral power, condition monitoring

## Abstract

An efficient maintenance is a key consideration in systems of railway transport, especially in high-speed trains, in order to avoid accidents with catastrophic consequences. In this sense, having a method that allows for the early detection of defects in critical elements, such as the bogie mechanical components, is a crucial for increasing the availability of rolling stock and reducing maintenance costs. The main contribution of this work is the proposal of a methodology that, based on classical signal processing techniques, provides a set of parameters for the fast identification of the operating state of a critical mechanical system. With this methodology, the vibratory behaviour of a very complex mechanical system is characterised, through variable inputs, which will allow for the detection of possible changes in the mechanical elements. This methodology is applied to a real high-speed train in commercial service, with the aim of studying the vibratory behaviour of the train (specifically, the bogie) before and after a maintenance operation. The results obtained with this methodology demonstrated the usefulness of the new procedure and allowed for the disclosure of reductions between 15% and 45% in the spectral power of selected Intrinsic Mode Functions (IMFs) after the maintenance operation.

## 1. Introduction

Railway researchers, manufacturers, and operators consider that ‘the economic efficiency and competitiveness of railway transport depends on the safety, availability and maintenance’ of each structural element that suffers high stress, as bogies or wheelsets [1]. The failure of one of these elements could have catastrophic effects for rolling stock and, particularly, for people, as happened in the accidents at Eschede (Germany) [2] in 1998 and Viareggio (Italy) [3] in 2009.

With this in mind, scientific research has aimed to reduce the number of accidents due to the failure of critical rolling elements [4,5]. For example, the EURAXLES project [6], a European project from 7th Framework Programme, ‘aims to bring the risk of failure of railway axles to such a minimum level that it will no longer be considered as a significant threat to the safe operation of the European interoperable railway system’ [6].

Moreover, the current complex and competitive railway market, which also involves the required maintenance activities for rolling stock, is encouraging the adoption of new and advanced inspection methods that are significantly different from traditional techniques [7]. These new methods are based on predictive techniques that allow for the condition diagnosis of critical elements of the rolling stock by studying failure-specific problems [8]. In addition, their deterioration can be predicted based on output variables. As result, the analysed mechanical elements save maintenance costs and increase their reliability [9,10].

Vibration analysis is one of the most used techniques for inspecting railway mechanical components in operation, as it allows for the conduction of tests on a wide range of elements, including railway infrastructure and rolling-stock [7]. Moreover, if this technique is combined with good supervising software, we can obtain a powerful tool for monitoring the condition of a critical element.

Most of the authors who study vibrations for condition monitoring in railway systems apply their techniques to infrastructure elements. Many of them have dealt with vibration analyses on railway systems, mainly focusing on the ground or track perturbations induced by the transit of rolling stock [11,12,13,14,15]. Some researchers [14,16,17,18] identify the track condition based on the vibration measures recorded by accelerometers that are mounted in the axle boxes of rolling stock. In fact, Wei et al. [19] developed a system for the online monitoring of track conditions.

Some authors apply vibration analysis to the rolling elements with the aim of inferring a change in the natural vibration modes [20] or a vibration pattern associated with known defects [21]. In this framework, real scale cracked axles were tested under different load conditions and crack sizes [4,22], as well as complete bogies of a Shinkansen train with deliberately generated defects [23]. However, all these tests were carried out in laboratory conditions.

The full-scale tests with rolling stock in an open track are extremely unusual, especially if we talk about in-service trains. In fact, Jeon et al. [24] recorded the vibration data from the axle boxes of a high-speed train running at very high speeds. However, these measurements were picked up from a prototype train in development under trial conditions. On the other hand, Trilla and Gratacòs [25] studied the behaviour of bogie components by analysing their vibration signature after maintenance. However, these tests were carried out using an underground train at low speed in depot-based test conditions. Another type of study is the approach to online condition monitoring performed by Papaelias et al. [26]. They suggest a method based on high-frequency acoustic emission and vibration analysis for monitoring the state of trains.

Some researchers have applied the Empirical Mode Decomposition (EMD) technique to vibration analysis [25,27,28,29]. Trilla and Gratacòs applied the EMD technique to an underground train in Reference [25] and established ‘that the second IMF was a reliable indicator of the condition of the axle box, and the third and fourth IMFs were reliable indicators of the condition of the wheelset’. Ahn et al. proposed, in Reference [28], the use of the proper orthogonal value (POV) of IMF as a potential parameter for monitoring the condition of rolling bearings. Yi et al. [29] developed a technique for the automatic and adaptive selection of IMFs on fault-bearing signals. The proposed technique allows for the diagnosis of railway bearings with different defects.

The main idea behind the EMD technique is the decomposition of vibration signals into sub-signals called Intrinsic Mode Functions (IMF). Theoretically, each IMF is directly related to the sources that generate it. In the present case, this is the mechanical components of the rolling stock. Therefore, the reason for applying the EMD technique in this work is to identify the vibratory contribution of each mechanical component to the total vibration through the decomposition of the vibration in several IMFs. Each IMF must belong to a specific mechanical element or a set of specific elements, so their quantification should be a good parameter for estimating the condition of the rolling stock, whose fault could result in human fatalities.

This work studies the dynamic behaviour of a high-speed train running gear [30] or bogie with the aim of determining a set of parameters based on IMF that allows for the fast identification of the running gear’s operating state. To that end, a high-speed train is equipped with an on-board measurement system that acquires vibration signals and then transmits them to a remote database. Tests are carried out before and after a scheduled maintenance activity without disturbing the regular operation of the train around the Spanish high-speed lines.

This paper is organised as follows: first, the methodology and fundamentals of applied methods are proposed. Next, the measurement system and the working scenario are described. Afterwards, the application of these techniques to the problem is presented and the obtained results are discussed. Finally, conclusions are drawn.

## 2. Proposed Methodology

**Figure 1:**
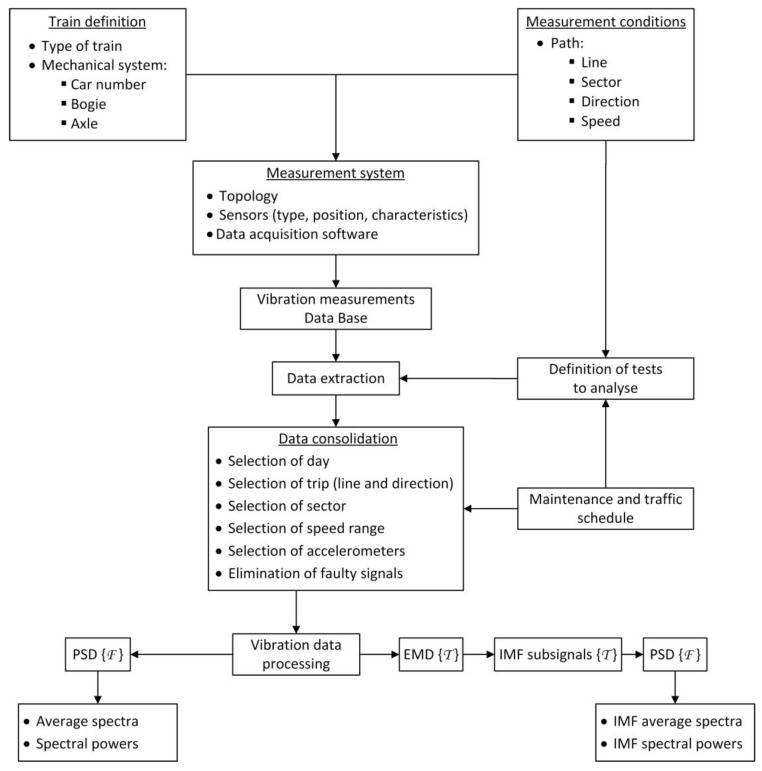
Diagram of the applied methodology.

This section describes the methodology and techniques that will be applied to the processing of vibration signals. The analysis process (see Figure 1) has two start points. The first one is the definition of the train under study, which implies the characterisation of the type of train and its specific mechanical system that is to be analysed. The second start point concerns the definition of the measurement conditions, the sector of the railway line, and the speed at which the tests will be carried out. Then, the main features of the measurement system installed on the train are determined based on these input data. The measurement equipment is set up to take measurements continuously and to record the data in a remote database.

Later, the vibration data are extracted and converted to a MATLAB^®^ format for signal processing. The selection of tests and the extraction of raw data from the database is made based on the measurement conditions, and the maintenance and traffic schedule document. The vibration data in the MATLAB^®^ format are pre-processed with the help of the maintenance and traffic schedule document. In this way, the vibration data for a specific day, sector, speed, and accelerometer are obtained. Then, if needed, the faulty signals are removed, which leaves a set of consolidated vibration data that are grouped by the accelerometer. Finally, the consolidated data are processed in the time and frequency domains, and the obtained results are analysed.

Once the vibration data are consolidated, they are processed. The first step (left branch of the vibration data processing, see Figure 1) is the computation of the Power Spectral Density (PSD) of every signal using Equation (1):

(1) $$S(f)=\frac{\Delta t}{N}{\left|X(f)\right|}^{2};\;PSD=S(f)$$

in which *∆t* is the sample time, *N* is the number of data points in the signal, and *X*(*f*) is the Fourier transform of the signal.

The average *PSD* ($\bar{PSD}$) of every set of vibratory signals grouped by accelerometer, railway line sector, and date is also computed. Equation (2) is used for this purpose:

(2) $$\bar{PSD}=\frac{\sum_{k=1}^{n_{s}}PSD_{k}}{n_{s}}$$

in which $\bar{PSD}$ is the average *PSD* of all processed signals, *PSD_k_* is the power spectral density of signal *k*, and *n_s_* is the number of recorded signals.

Then, the spectral or signal power is computed. By the definition presented in Reference [31], the signal power *P* equals the integral (or sum, for the discrete case) over the frequency range of the general distribution function *S* or *PSD*, which leads to Equations (3) and (4).

(3) $$P=\sum_{f=0}^{N-1}S(f)\Delta f=\sum_{f=0}^{N/2}S_{one}(f)\Delta f$$

(4) $$S_{one}(f)=\left\{ \begin{array}{l} 2S(f)\Rightarrow f=1\ldots \frac{N}{2}-1\\ S(f)\Rightarrow f=0,f=\frac{N}{2} \end{array}\right.$$

in which *∆f* is the frequency interval, *N* is the number of data points in the signal, and *S*(*f*) is the *PSD* of the signal.

In the right branch of the vibration data processing (see Figure 1), the EMD technique is applied to every analysed vibration signal. The main idea behind this technique is to identify the intrinsic oscillatory modes of a given dynamic signal by their characteristic time scales and decompose the data according to these modes [32]. In practice, this technique is an iterative method for decomposing a dynamic signal into a set of IMFs, which is based on three assumptions:

- In the given signal, the number of extrema is at least two: one maximum and one minimum.
- The characteristic time scale is defined by the time lapse between the extrema.
- If the given signal does not have extrema but does have inflexion points, the data can be differentiated to disclose the extrema.

The algorithm to obtain the IMF, or Intrinsic Mode Function, of a given signal *x*(*t*) implies two loops. The inner loop is known as the sifting process. This loop runs until the extracted signal meets the IMF conditions [32]: (1) In the whole data set, the number of extrema and the number of zero-crossings must either equal or differ at most by one; and (2) At any point, the mean value of the envelope defined by the local maxima, and the envelope defined by the local minima is zero.

The outer loop extracts all the intrinsic mode functions of the input signal. The whole algorithm works as follows:

1. Identify all the extrema of a given signal *x*(*t*).
2. Connect all the maxima of a given signal *x*(*t*) with a cubic spline curve, obtaining an upper envelope *e_max_*(*t*). Repeat the process connecting all minima of a given signal *x*(*t*), which gives us a lower envelop *e_min_*(*t*). Compute the mean between the envelopes resulting *m*_1_(*t*) as Equation (5).
  (5) $$m_{\;1}(t)=\frac{e_{\max}(t)+e_{\min}(t)}{2}$$
3. Subtract the mean from the input signal to obtain the first IMF candidate *h*_1_(*t*) as Equation (6).
  (6) $$h_{1}(t)=x(t)-m_{\;1}(t)$$
4. If *h*_1_ does not meet the IMF conditions, take *h*_1_(*t*) as the input data, and repeat steps 1–3 until *h*_1*n*_(*t*) meets the IMF conditions, following the procedure of Equation (7).
  (7) $$h_{1n}(t)=h_{1(n-1)}(t)-m_{\;1}(t)$$
5. When *h*_1*n*_ meets the IMF conditions, it is renamed as *c*_1_ and becomes the first IMF component from the data.
6. Calculate the residue (see Equation (8)).
  (8) $$r_{1}(t)=x(t)-c_{1}(t)$$
7. Use the residue as the new input signal, and repeat steps 1–6 until no more IMFs can be extracted from the new input signal; then, follow the procedure given by Equations (9) and (10).
  (9) $$c_{k}(t)=r_{k-1}(t)-m_{k}(t)$$
  (10) $$r_{k}(t)=r_{k-1}(t)-c_{k}(t)\;(\;r_{0}(t)=x(t)\;)$$

In the end, the input signal *x*(*t*) is decomposed into the addition of a set of *N_E_* components *c_k_*(*t*) or IMF and the residue (see Equation (11)).

(11) $$x(t)=\sum_{k=1}^{N_{E}}c_{k}(t)+r_{N}(t)$$

In this research, we use the bivariate EMD algorithm developed by Rilling et al. [33] to obtain the IMF. The idea behind this algorithm is the one described above but, in addition, it gives us the ability to perform decompositions of complex signals.

Once the IMFs are obtained, a set of EMD-based parameters is computed. For that purpose, the average *PSD* and the spectral power of each extracted IMF per accelerometer is calculated. We can modify Equation (1) for each extracted IMF, obtaining Equation (12):

(12) $$S^{c_{k}}(f)=\frac{\Delta t}{N}{\left|X^{c_{k}}(f)\right|}^{2};\;PSD^{c_{k}}=S^{c_{k}}(f)$$

in which *∆t* is the sample time, *N* is the number of data points in the signal, *X^c_k_^*(*f*) is the Fourier transform of the IMF *c_k_*(*t*), and *S^c_k_^* (or *PSD^c_k_^*) is the power spectral density of IMF *c_k_*(*t*).

The average *PSD* ($\bar{PSD}$) of every set of IMF grouped by accelerometer, railway line sector, and date was also computed. Equation (13) is used for this purpose:

(13) $${\bar{PSD}}^{c_{k}}=\frac{\sum_{j=1}^{n_{s}}PSD_{j}^{c_{k}}}{n_{s}}$$

in which ${\bar{PSD}}^{c_{k}}$ is the average *PSD* of the processed IMF, *PSD^c_k_^* is the power spectral density of the IMF *c_k_*(*t*) extracted from the *j*th signal *x*(*t*), and *n_s_* is the number of recorded signals. Finally, the spectral power of each IMF is computed by applying Equations (3) and (4).

## 3. Experimental System

The proposed methodology is applied to a high-speed train in order to analyse the behaviour of its rolling elements after a wheels intervention. To that end, in-service measurements are recorded and compared before and after the maintenance tasks.

The experimental system used in this work is designed to take vibration measurements without disturbing the standard operation of the train. This system is installed in a high-speed train so that the vibration signals are recorded at speeds up to 300 km/h. The train is composed of two power cars at the ends of the train and eight intermediate passenger cars that accommodate more than 300 people. This type of train follows an extensive maintenance schedule that goes through a deep preventive work every month and wheel interventions 3–4 times per year.

The train under study is equipped with a vibration measuring system, a data acquisition system, and a communication system. The monitored train is usually assigned to operate journeys on two high-speed lines, which will be called HSL-A (high-speed line A) and HSL-B (high-speed line B) due to confidentiality requirements of the companies involved in this project.

In order to study the dynamic behaviour of this train, a 150 km-length sector of the HSL-A, in which the train runs at an average speed of 270 km/h (close to the maximum speed of the railway line), is selected.

The length of the studied railway sector provides a sufficient number of vibration signals, and the fact the train travels at an almost constant speed gives us a high uniformity in its dynamic behaviour. With the aim of reducing (as far as possible) the unknown parameters, two journeys in similar conditions (the same railway line, direction, sector, speed, time, train, bogie, and wheelset) are selected, one before and one after the maintenance action.

**Figure 2:**
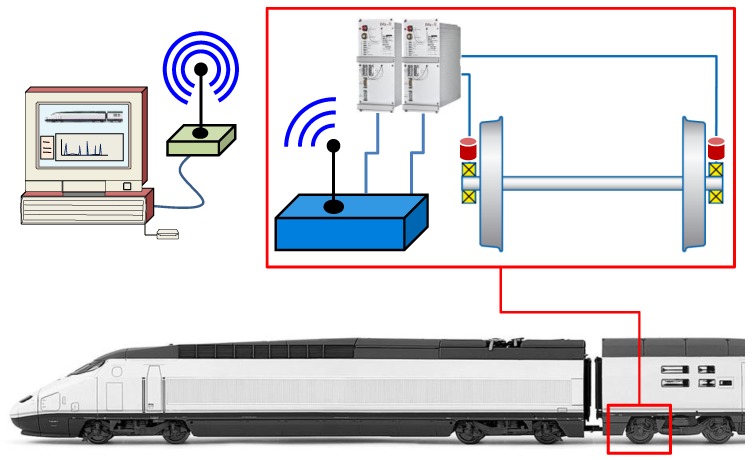
Diagram of the whole measurement system.

The whole measurement system (vibration measuring, data acquisition, transmission, and recording systems) is composed of two main parts: (1) an on-board measurement system installed inside the luggage compartment of the last passenger car, and (2) a database located at the MAQLAB Laboratory in the Universidad Carlos III de Madrid. The diagram of the measurement system is shown in Figure 2.

The on-board measurement system comprises of a DC power supply unit, two IMx-R units for data acquisition, a UMTS (3G) router for data transmission, a speed sensor, and three uniaxial accelerometers.

**Figure 3:**
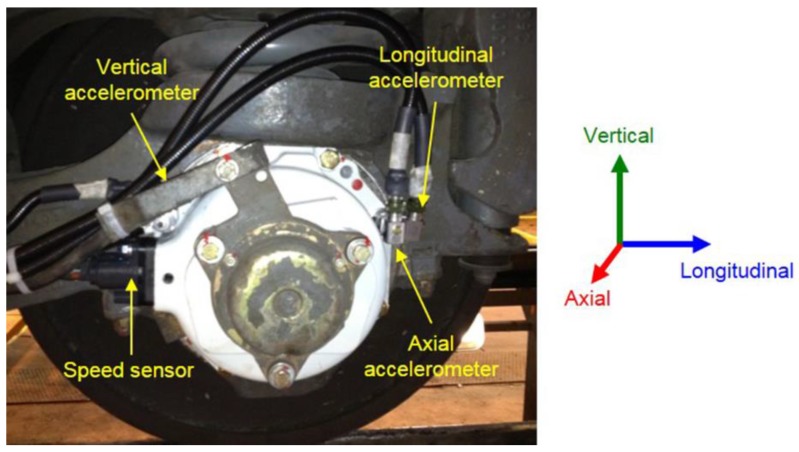
The location of the longitudinal, axial, and vertical accelerometers and speed sensor after their installation in the axle box.

The accelerometers and the speed sensors are mounted in the axle box cover of a trailer axle. This axle is mounted in a bogie of the last passenger car and is the nearest axle to the second power car. The three uniaxial accelerometers are arranged to measure the acceleration in the three directions of space (longitudinal, axial, and vertical) and are installed on the axle box cover. Unlike the accelerometers, the speed sensor is embedded inside the axle box (see Figure 3). By placing the measurement point in the axle box cover, it is possible to acquire the vibration signal from the bearing and from the wheelset (which involves the axle, the wheels, and the brake disks). The accelerometers must be reliable, withstand severe climatic conditions, and have a large enough measurement range for the nature of the vibrations expected in a common commercial journey of the train. It is for all these reasons that ICP accelerometers of industrial use with a measurement range of ±50 g, a frequency range from 0.52 Hz–8 kHz, and a sensitivity of 100 mV/g have been selected.

The equipment is set up to acquire acceleration signals in a speed range between 75 and 2000 rpm. When the train runs at its maximum speed of 300 km/h, the angular speed is 1730 rpm, so the selected range is large enough to meet the normal operation of the monitored train. The sampling rate is established at 5120 Hz, and the sampling time is set at 3.2 s. This implies 16,384 data points per measure, saving the waveform of the signal in the remote database.

The axle box mounts a double row tapered roller bearing in a TDO configuration that is specifically designed for high-speed trains.

The database located at the MAQLAB Laboratory is built on an SQL Server^®^ and is managed through a condition monitoring software. This software stores the acceleration and the speed data received from the on-board measurement system in the database, as well as the date, accelerometer identifier, and other useful information.

The rolling bearings have characteristic fault frequencies that are interesting to know in order to detect faults in the frequency spectrum. These values are calculated in advance according to Equations (14)–(17) developed by Martin in Reference [34]:

(14) $$BPFI=\frac{N_{b}}{2}F_{s}\left(1+\frac{d}{D}\cos\beta \right)$$

(15) $$BPFO=\frac{N_{b}}{2}F_{s}\left(1-\frac{d}{D}\cos\beta \right)$$

(16) $$BSF=\frac{D}{2d}F_{s}\left[1-{\left(\frac{d}{D}\right)}^{2}\cos^{2}\beta \right]$$

(17) $$FTF=\frac{F_{s}}{2}\left(1-\frac{d}{D}\cos\beta \right)$$

in which *F_s_* is the axle rotating frequency, *N_b_* is the number of rolling elements, *d* is the rolling element diameter, *D* is the pitch diameter, and *β* is the contact angle. The actual calculated values of fault frequencies for a travel speed of 270 km/h (the average travel speed in the analysed railway sector) are shown in Table 1.

## 4. Results

This section summarises the results obtained from the application of the methodology described above to the vibration signals acquired by the on-board experimental system. With the aim of studying the influence of a maintenance activity (consisting of a wheel reprofiling) in the dynamic behaviour of the train, two different operating states are analysed: one before the maintenance task and one after the wheels intervention.

The maintenance activity focused on a wheel intervention that consisted of reprofiling the two wheels of the axle. The turning of the wheel regains its original thread profile and eliminates defects such as wheel corrugation. The difference between the nominal diameters before and after the wheel reprofiling is 3 mm.

The average PSD is computed by taking into account all recorded signals (in each journey and spatial direction) and plotted in pairs to facilitate the comparison between the two studied operating states (before and after the maintenance operation).

**Figure 4:**
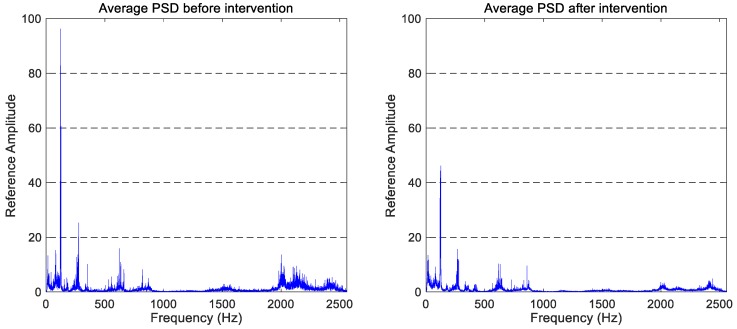
The comparison of the average spectra for vertical accelerations obtained before and after the wheel intervention.

The average PSD graphs that correspond to the vertical vibration signals in the two states studied (after and before wheel intervention) are shown in Figure 4. Due to confidentiality reasons of the companies involved in this project, the *y*-axis of the figures has been referenced to the full scale of the ‘before maintenance’ spectra.

The comparison between both vertical spectra shows a general reduction in the vibration signature, which allows us to confirm that the maintenance work has an observable effect on the monitored wheelset. More precisely, the reduction of the vibration level in the 100–300 Hz and 1750–2500 Hz frequency ranges can be highlighted. The latter is consistent with the wheel reprofiling, as that frequency band matches the frequency range of wheel corrugation according to Kouroussis et al. [35].

Preliminary studies of spectra and average spectra of several journeys show repetitive frequency components and active zones in all spectra. Taking the vertical vibration signals as an example—because the vertical spectra are the cleanest—six active zones can be identified and named as follows:

- The A-band comprises of the 0–100 Hz frequency range.
- The B-band includes the 100–350 Hz frequency region.
- The C-band comprises of the 350–700 Hz frequency range.
- The D-band involves the 700–1100 Hz band.
- The E-band encloses the small active region located in the 1100–1700 Hz frequency range.
- The F-band is located in the high-frequency area, above 1700 Hz.

**Figure 5:**
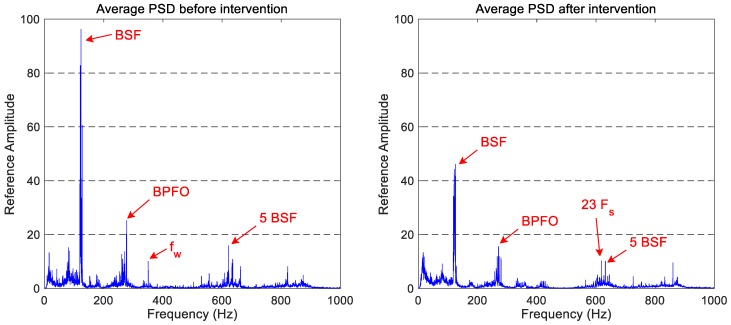
The comparison of the average spectrum for vertical accelerations obtained before and after the wheels intervention, in the 0–1000 Hz frequency range.

The most active frequency components in both vertical spectra of Figure 4 are located in the 0–1000 Hz frequency range. So, if we focus on this frequency band (see Figure 5), the main spectral components can be identified. The main peak is located around 125 Hz, which coincides with the ball spin frequency (BSF) of the bearing. There is also a component around 625 Hz, which corresponds to the five-multiplier of the BSF. Another interesting peak is located at 275 Hz, which matches the BPFO of the bearing.

There is also an interesting peak at 350 Hz. This frequency component coincides with the first vibration mode of the wheel (identified by *f_w_* in Figure 5) and only appears before the maintenance task.

**Figure 6:**
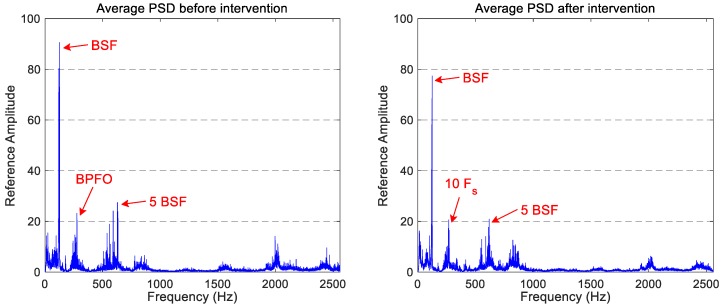
The comparison of the average spectrum for longitudinal accelerations obtained before and after the wheels intervention.

**Figure 7:**
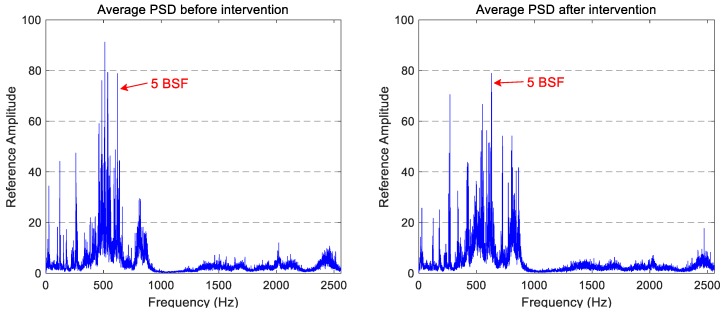
The comparison of the average spectrum for axial accelerations obtained before and after the wheels intervention.

The average PSDs for longitudinal and axial vibration signals are shown in Figure 6 and Figure 7, respectively. In the longitudinal vibration signal spectra, a small decrease in the vibration signature of the component located at approximately 125 Hz (the component that coincides with the BSF) and of the high-frequency region is observed. The reminder spectrum keeps the same power level or undergoes a small reduction of power.

However, the axial vibration signal spectra show several peaks with a chaotic performance: some components reduce their power level, whereas others increase it. This does not allow for the establishment of an observable influence on the maintenance task in the vibration signature of the axial accelerometer. In both cases, the characteristic components identified in the vertical spectra (BSF, BPFO, and the five-multiplier of BSF) are also visible in the longitudinal and axial spectra.

The spectral power values of the vibration signals are computed before and after the maintenance operation, which consists of a wheel intervention. The results obtained in this process are collected together in Table 2 for better comparison. A significant reduction of the spectral power of vertical vibrations by 25.20% is visible. However, the spectral power of the longitudinal vibration maintains the same level as the power is reduced by only 0.09%. On the other hand, the spectral power of axial vibrations undergoes an increase of 2.46%. Nevertheless, variations below 10% cannot be considered as significant in the studied system and test conditions. These numerical results quantify what is observed in spectra graphs and make clear that the wheel intervention especially affects the vertical dynamics of the rolling stock.

**Figure 8:**
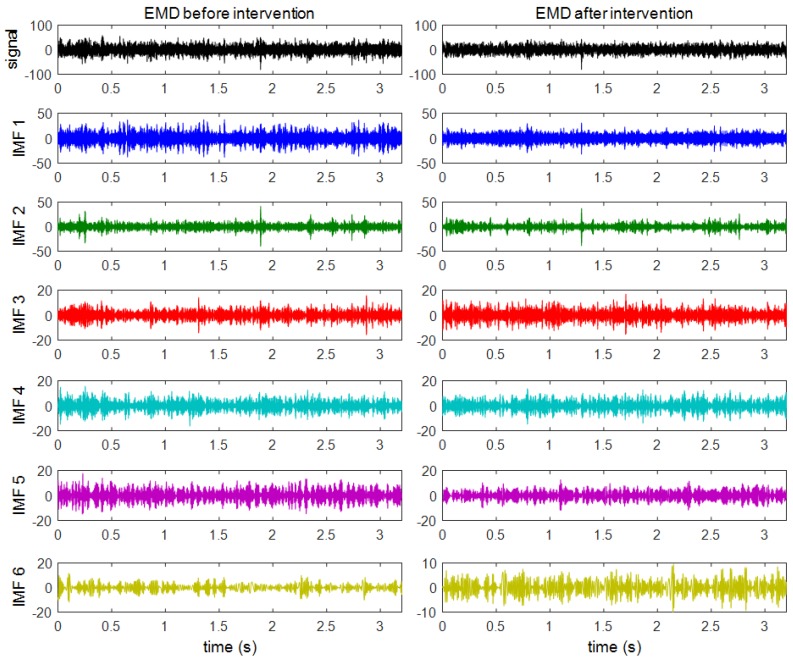
The original signal (in black) and the first six IMF (in different colours) of vertical vibration signals before and after maintenance.

In order to identify the six active zones (defined above) with the vibration of a specific mechanical component, the vibration signals will be decomposed in six IMFs as stated in the methodology. The decomposition in several IMFs will also allow for the establishment of the best IMFs as indicators of the operating state of the bogie. Figure 8 shows the original signal and the first six IMFs obtained after the application of the EMD technique to two vertical vibration signals. The vibration signals before maintenance are shown on the left and the vibration signals after maintenance are shown on the right.

**Figure 9:**
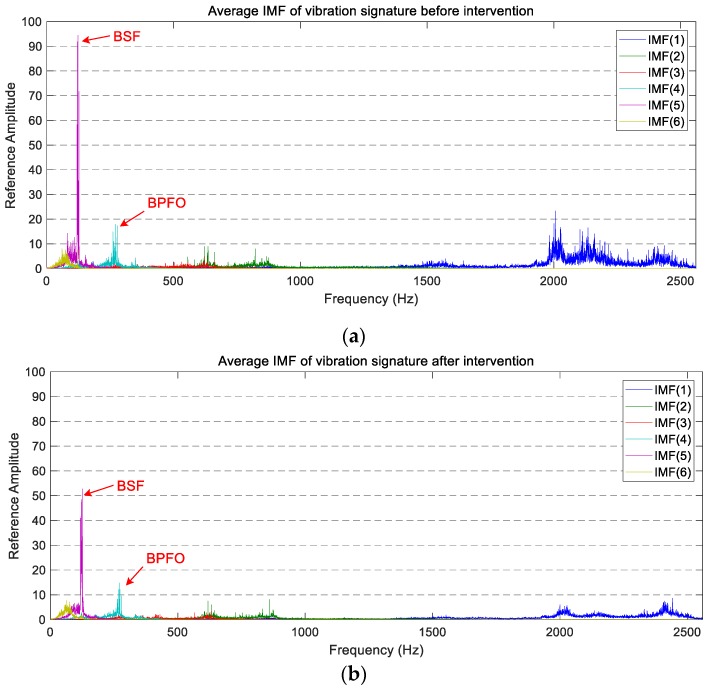
The average PSD of the IMFs corresponding to the vertical vibration signals: (**a**) before maintenance; (**b**) after maintenance.

Once the IMFs are obtained, the average spectra of the six IMFs of the two operating states are computed by applying Equation (13). The average spectra of the six IMFs corresponding to the vertical measurements in both states are shown in Figure 9. For better understanding, the average PSD of each IMF has been plotted using the same colour code as in Figure 8.

By comparing the six active zones of the PSD described above with the spectra of the IMF, it is possible to relate the active zones and the IMF. Approximately, IMF(1) links to the F-band and the E-band, IMF(2) connects to the D-band, IMF(3) relates to the C-band, IMF(4) and IMF(5) link to the B-band, and the IMF(6) is associated with the A-band. 

In general, the appearance of the IMFs’ vertical spectra is like the PSD’s vertical spectrum exposed above. That is, the most active area is located above 1700 Hz and two significant components exist near 125 Hz and 275 Hz.

By studying each IMF, a significant reduction in the power level of IMF(1), IMF(4), and IMF(5) is visible after the wheel intervention was carried out. This reduction is especially relevant in the main peak of the IMF(5), which coincided with BSF and where the amplitude reduction is about 45%. The IMF(1) also has a capital interest, as it undergoes a big amplitude reduction, and it is in the wheel corrugation frequency, which could relate it directly to the wheel condition. The vibration level of the other three IMFs remains at a similar level after maintenance.

**Figure 10:**
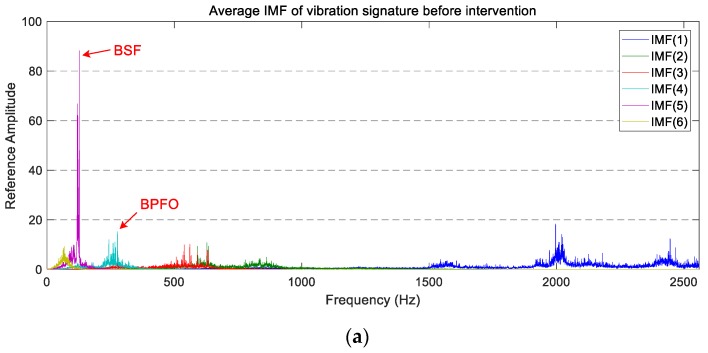

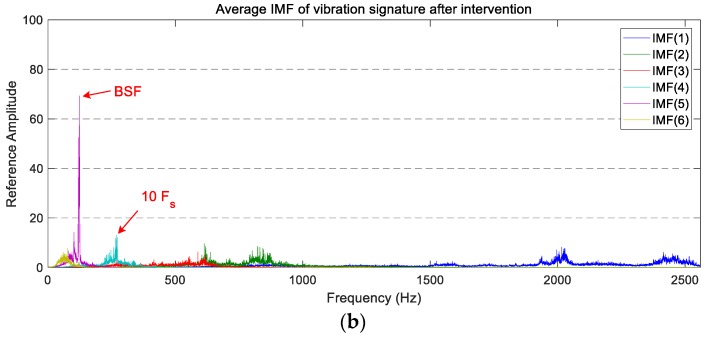
The average PSD of the IMFs corresponding to the longitudinal vibration signals: (**a**) before maintenance; (**b**) after maintenance.

The average PSD of the vibration signatures of longitudinal IMFs are displayed in Figure 10. In this case, a reduction in the power level of IMF(1), IMF(3), and IMF(5) is observable after the wheel intervention. On the other hand, IMF(4) and IMF(6) present a similar vibration level before and after maintenance, whereas IMF(2) presents higher peaks between 700 Hz and 1000 Hz.

**Figure 11:**
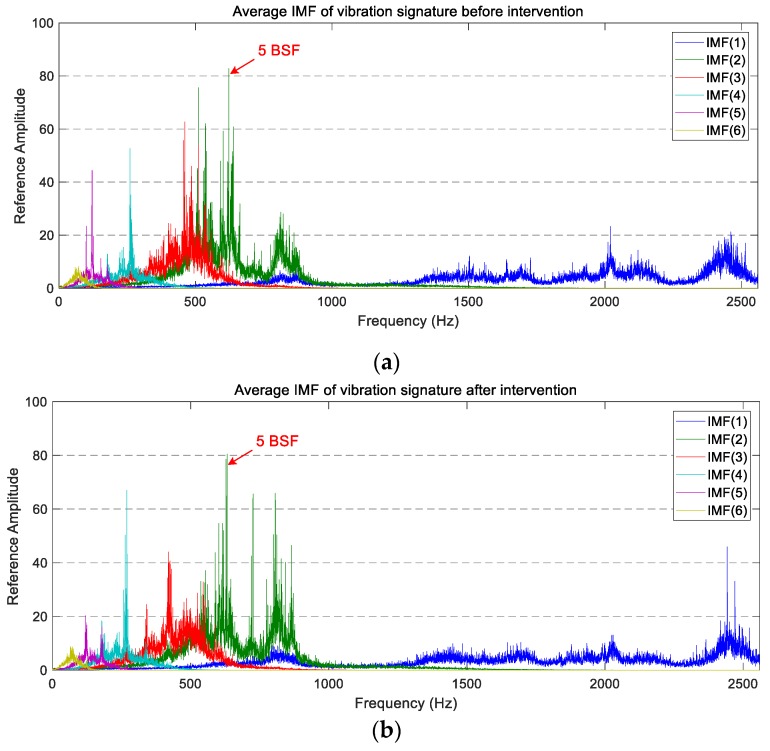
The average PSD of the IMFs corresponding to the axial vibration signals: (**a**) before maintenance; (**b**) after maintenance.

By analysing the decomposed vibration signatures of the axial accelerometer (whose average PSDs are plotted in Figure 11), the same chaotic behaviour observed previously in the average PSD is visible. The individual comparison of IMFs before and after maintenance work does not allow for the extraction of a defined trend. This is because the overall vibration level and the magnitude of the highest peaks are similar in both operating states.

**Figure 12:**
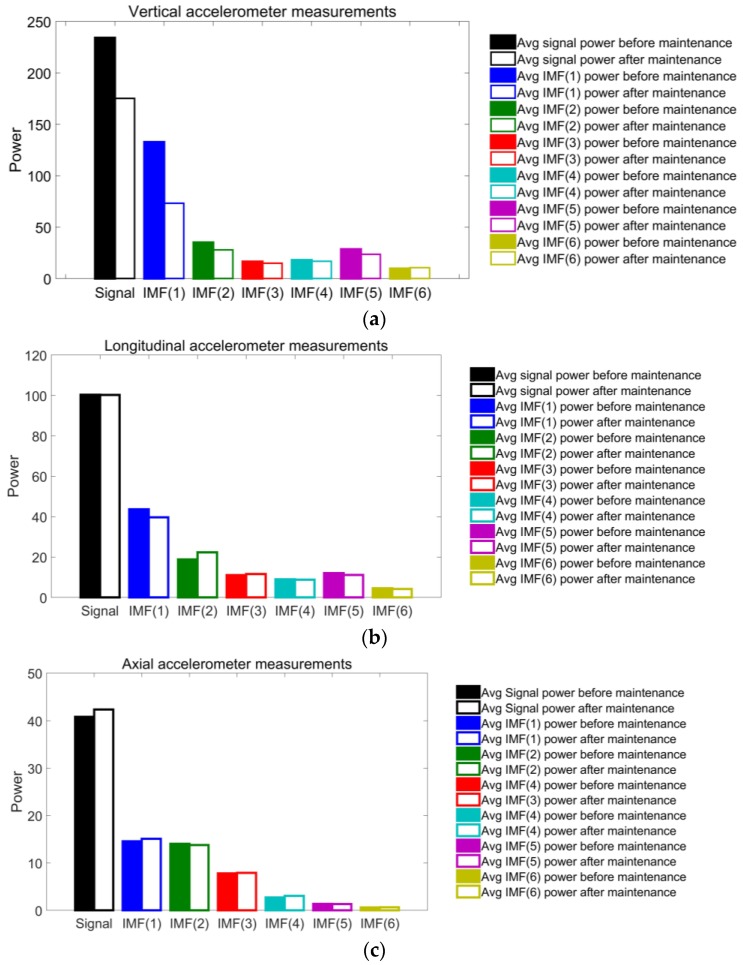
The average spectral power values of the signal and the IMF of (**a**) the vertical; (**b**) the longitudinal, and (**c**) the axial vibrations before (filled bars) and after (blank bars) wheel intervention.

The spectral power values of each IMF in the three vibration directions are calculated and plotted in the bar charts of Figure 12. The spectral power of the average PSD is also plotted and identified as ‘signal’. In the vertical data (see Figure 12a), the vibration level decreases in all IMFs, except in IMF(6), in which it growths slightly. The reduction is especially notable in IMF(1), IMF(2), and IMF(5), with reductions of 44.83%, 20.01%, and 16.91%, respectively. However, the great reduction observed in the main peak of IMF(5) is not seen by applying this technique.

Concerning the longitudinal data (see Figure 12b), the spectral power values are reasonably stable. Four IMFs reduce their spectral power, while the other two increase it. However, these variations do not reach 10%. The only exception is IMF(2), which undergoes an increase of 18.9%. The greatest decrease occurs at IMF(1), with a power reduction of 9.23%.

The spectral power values of the axial vibration (see Figure 12c) keep the same level before and after the maintenance tasks. Three IMFs increase the power level, and the other three decrease it. All variations but one are in the range of ±3.5%, and the biggest takes place on IMF(4), which suffers a reduction of 7.63%.

## 5. Conclusions

In this paper, a new methodology for analysing railway vibration data is proposed. It is based on the quantification of the differences in the average spectral powers of two operating states in order to identify the condition of the monitored mechanical system. This methodology is focused on the vibration analysis and operating condition identification of a railway bogie, although it is applicable to any other mechanical system as well.

The average spectra are obtained from vibration signals in the three directions of space. The spectra present several active zones common to the three directions of the accelerometers, although they vary in their limits.

The vertical vibrations are highly influenced by the condition of the wheelset. By examining the spectrum after the wheel reprofiling, a great reduction in the high-frequency range (above 1700 Hz and coincident with the wheel corrugation frequency range [35]) and in the components located at 125 Hz and 275 Hz—which approximately matches the fault bearing frequencies—was visible. The spectral power of the vertical vibration undergoes a 25% power reduction after the maintenance task. The longitudinal and axial vibrations do not present significant trends before and after the wheel intervention.

The application of the EMD to collecting the vibration signal allows for the in-detail identification of the contribution of different phenomena to the vibrational behaviour of the train. We can relate IMF(1) to the wheel corrugation phenomenon, and IMF(4) and IMF(5) to bearing faults. In addition, these IMFs undergo a noteworthy vibration level reduction in the vertical spectrum. The longitudinal and axial spectra of IMFs present similar vibration levels before and after maintenance.

The reductions of 44.83%, 20.01%, and 16.91% of IMF(1), IMF(2), and IMF(5), respectively, in their vertical spectral powers, make these IMFs good indicators for estimating the condition of the high-speed train running gear system. The slight variation of the power in the longitudinal and axial directions does not allow for them to be established as indicators of the bogie condition.

As a final conclusion, the proposed methodology was demonstrated to be a useful tool for the identification of the bogie operating state of a high-speed train in service. Most importantly, the main parameters that were used to monitor the operating state of the bogie consisted of the total spectral power and the IMF(1), IMF(2), and IMF(5) spectral power of the vertical vibrations.

## Figures and Tables

**Table 1:** The bearing characteristic frequencies at 270 km/h.

Axle or Bearing Parameter and Faults	Characteristic Frequency (Hz)
Axle rotation frequency (*F_s_*)	26.48
Ball Pass Frequency Inner Race (*BPFI*)	335.87
Ball Pass Frequency Outer Race (*BPFO*)	273.17
Ball Spin Frequency (*BSF*)	125.36
Fundamental Train Frequency (*FTF*)	11.89

**Table 2:** The spectral power values for each accelerometer before and after wheel intervention.

Accelerometer	Before	After	Difference (%)
Vertical	234.15	175.15	−25.20
Longitudinal	100.38	100.29	−0.09
Axial	40.83	41.84	2.46
